# Development and Evaluation of a Virtual Environment to Assess Cycling Hazard Perception Skills

**DOI:** 10.3390/s21165499

**Published:** 2021-08-16

**Authors:** Kjell van Paridon, Matthew A. Timmis, Shabnam Sadeghi Esfahlani

**Affiliations:** 1 Cambridge Centre for Sport and Exercise Sciences (CCSES), School of Psychology and Sport Science, Anglia Ruskin University, Cambridge CB1 1PT, UK; matthew.timmis@aru.ac.uk; 2 Medical Technology Research Centre (MTRC), School of Engineering and Built Environment, Anglia Ruskin University, Essex CM1 1SQ, UK

**Keywords:** virtual reality, serious video game, visual search, cycling, hazard perception

## Abstract

Safe cycling requires situational awareness to identify and perceive hazards in the environment to react to and avoid dangerous situations. Concurrently, tending to external distractions leads to a failure to identify hazards or to respond appropriately in a time-constrained manner. Hazard perception training can enhance the ability to identify and react to potential dangers while cycling. Although cycling on the road in the presence of driving cars provides an excellent opportunity to develop and evaluate hazard perception skills, there are obvious ethical and practical risks, requiring extensive resources to facilitate safety, particularly when involving children. Therefore, we developed a Cycling and Hazard Perception virtual reality (VR) simulator (CHP-VR simulator) to create a safe environment where hazard perception can be evaluated and/or trained in a real-time setting. The player interacts in the virtual environment through a stationary bike, where sensors on the bike transfer the player’s position and actions (speed and road positioning) into the virtual environment. A VR headset provides a real-world experience for the player, and a procedural content generation (PCG) algorithm enables the generation of playable artifacts. Pilot data using experienced adult cyclists was collected to develop and evaluate the VR simulator through measuring gaze behavior, both in VR and in situ. A comparable scene (cycling past a parked bus) in VR and in situ was used. In this scenario, cyclists fixated 20% longer at the bus in VR compared to in situ. However, limited agreement identified that the mean differences fell within 95% confidence intervals. The observed differences were likely attributed to a lower number of concurrently appearing elements (i.e., cars) in the VR environment compared with in situ. Future work will explore feasibility testing in young children by increasing assets and incorporating a game scoring system to direct attention to overt and covert hazards.

## 1. Introduction

Cycling is a suitable strategy to increase physical activity levels [1]. Whilst bicycle ownership is high [2], the uptake of cycling for transport or leisure in both adults and children is low. This uptake is partly attributed to the risk perception associated with the dangers of cycling [3]. Although objective dangers, such as road design and traffic density, are essential factors in determining risk perception, it is equally important to consider the ability to identify, anticipate, and adapt behavior in these dangerous situations. This process is commonly referred to as hazard perception.

Hazard perception can be defined as the ability to anticipate dangerous situations based on perceptual evidence [4]. Research suggests that expertise differences in hazard perception range from solely identifying salient information (e.g., cars) to “reading” more complex travel scenes to evaluate potential dangerous scenes of a less salient nature (e.g., considering not having a line of sight and covert hazards, [5,6]). These hazard perception abilities are typically examined in video-based paradigms, where reaction times, response rates, and eye movements from a road user’s perspective are investigated.

For example, the authors in [7] developed a 2-D cycling simulator and identified that young adult cyclists looked more at task-irrelevant objects in the environment compared to elderly adults. Similarly, researchers in [8] exposed young children to overt and covert hazards in a video-based paradigm to examine hazard identification and demonstrated that children had delayed reaction times and time until the first fixation on the latent covert hazards compared to adults.

While using 2-D video-based approaches to measure hazard perception in cyclists allows the direction of overt visual attention (i.e., gaze behavior) in identifying potential hazards to be measured, the initiation of avoidance strategies in response to these hazards is not commonly assessed. In addition, gaze behavior in 2-D video-based stimulation is adapted due to a reduction in contextual information [9], and participants’ adaptive behavior (e.g., avoidance behavior) is often not possible to examine.

The use of a stereoscopic VR environment provides further contextual information (i.e., depth), allowing enhanced perception of information, particularly if combined with physical interactivity in this environment. For example, the authors in [10] compared visual search behavior in real-world walking to navigating a virtual simulation of this environment. However, differences in gaze distribution were observed potentially due to the restricted physical movement in VR. There were no significant differences in dwell times between VR and the real world.

These findings support the use of VR as a simulation of the real world, particularly as it can enhance experimental control over real-world experiments while maintaining ecological validity. In our previous work, [11], we examined the visual search behavior of children cycling through naturalistic environments of various task complexities to understand children’s hazard perception abilities better. However, the lack of control in stimuli exposure made it challenging to examine hazard perception changes as a function of ability, cycle training, or hazard perception training (c.f., [12]). The development of a VR environment could provide opportunities to examine this while reducing the ethical and practical risks associated with the on-road assessment of cycling behavior in children and adults.

### Contributions

In the current work, we developed a Cycling and Hazard Perception VR simulator (CHP-VR simulator). The VR simulator includes a safe and controlled environment that monitors cyclist behavior while cycling in traffic and cluttered areas [13] (a busy environment with many distractions, e.g., people and street objects). The CHP-VR simulator reads real-time data from sensors while the player interacts with the environment. The developed framework aids in determining the cyclist’s reaction time in a hazardous situation, visual search behavior when encountering an obstacle/hazard, and managing the various situations (e.g., initiating an avoidance strategy).

Sensors were used to record the player kinematics, body orientation, and speed on the stationary bike, which was transferred to the virtual scene, enabling the player to proceed in the 3D world through a data-driven approach. Concurrently, visual orienting in VR was assessed by integrating eye-tracking with the VR head-mounted display to collect visual search data.

Virtual objects are generated using a procedural algorithm to offer an immersive environment that is replayable. The fusion of Procedural Content Generation (PCG) and Machine Learning (PCGML) algorithms facilitates a cost-effective optimum computation time for content generation in a VR environment. Optimum computation time is vital in this development due to the demand for real-time data from sensors to progress the virtual environment. Advancements in processing speed and graphics hardware made it possible to achieve a 3D environment that runs smoothly acquiring real-time messages from sensors generated by the player.

In this study, the use of PCGML rendered a unique appearance and performance with various hazardous conditions on streets and roads. The virtual hazards were generated via Non-Player Characters (NPCs) and objects, including pedestrians crossing a road, pedestrians congregating on the side of the road, a bench or bus stop, cars pulling over, or parking in cluttered areas that distract the player’s attention or obscures their view. The study uses the generated virtual environment to analyze cyclists’ field of view and visual fixation.

Pilot data was collected on older adults and demonstrated that it is possible to develop a CHP-VR simulator that reflects cycling in an outdoor environment. The results also indicate that cyclists behave comparably in VR compared to in situ environments. However, further work is needed to fully understand the comparability of visual search behavior in both VR and situ settings.

The aim of the study was two-fold. First, the aim was to develop an interactive and immersive CHP-VR simulator that demonstrated potential applicability for developing hazard perception among young cyclists. Based upon the successfully developed CHP-VR environment, the study’s second aim was to evaluate the behavior of cyclists in VR and in situ cycling.

## 2. Materials and Methods

### 2.1. Content Generation Algorithms

The virtual environment developed is interactively explored from a first-person perspective. All geometrical components of the streets are generated as the user encounters them. The scene and roads are generated using pseudo-infinite virtual objects in real-time [14]. A pseudo-irregular number generator is utilized with an integer derived from buildings, trees, and roads. The road plan is made by consolidating randomly generated geometries in an iterative interaction to foster intersections with sharp bends.

PCG’s critical property describes the entity as a sequence of generation instructions rather than static block information. The boundaries set in the algorithm allow the generation of occasions with differing qualities.

In a PCG, mathematical and texture data are not determined in the ordinary sense. Instead, details are abstracted into an algorithm with few details. The parameters in the algorithm are adjusted according to a specific behavior, i.e., the number of PCG segments. PCG enables capturing the essence of an entity without explicitly bounding it inside the present reality limit. By fluctuating boundaries, we could deliver a broad scope of results not restricted to the original model’s limitations.

To generate the terrain, we isolated it into square cells on a 2-D matrix where every cell addresses an intermediary for its procedurally produced content. The cells are orchestrated in square loops around the camera’s position situated at the middle. Cells are tried for possible permeability before their substance is produced. Every cell in our virtual scene contains buildings, roads, and trees. The point dictates the possible permeability between the cell and the viewing direction and the camera’s distance. In our execution, only the substance of cells situated inside a 120${}^{\circ }$ survey point and a distance of [loops × cell-size] are considered apparent.

The street’s appearance was determined by a single thirty-two-bit pseudo-random number generator (PRNG) seed [15]. The random number sequence creates streets with buildings, trees, and road layouts through the vertexes and height values. Comparative initial groupings of arbitrary numbers for comparable seeds were found with the irregular number generator. Comparative arrangements of numbers could bring about comparable formats. The terrain types are stored in a macro-matrix grid, which is utilized to make customized height maps.

If the vertex being analyzed is mapped to a macro-matrix location described as a green area with trees, a pedestrian crossing, buildings, or roads with traffic lights, then the algorithm assigns a height value required by that vertex to create the height. PRNGs produce a sequence of random numbers that are two-dimensional polygons with an initial seed value. When initialized with the same seed, indistinguishable groupings of numbers are delivered.

The subsequent virtual items have their stature and width characterized by the most extreme size of marked integers. The macro-matrix has (i, j) segments that address a few vertices in the virtual world. The terrain height generation uses parametric functions that inform the height value of each vertex. The functions are seeded with the point location in the world. Consequently, the function can describe all the height information globally with no limitation concerning its size. The processing time is identified with the user’s view as the functions employ the point information to calculate its height value.

Simplex noise (SN) stochastic functions are integrated to generate a pseudo-random gradient vector at each lattice point of the terrain, and the pseudo-random gradients determine its behavior between lattice points and interpolate a smooth function between the points. The method comprises making a lattice of irregular slopes, the dot products of which are then interpolated to acquire values between the grids [16]. Each corner of the SN containing *P* has a unit length random gradient vector $G_{v}c=G\left(P\right)$ where *G* is the pseudo-random gradient function and *c* is one of the N+1 corners of the SN.

The scalar item is found between the inclination vector $G_{v}c$ and the gradient slopes $G_{r}c$ to acquire a scalar value of $S_{c}$. In SN, each $S_{c}$ esteem is increased by a radially symmetric lessening function to obtain a weighted scalar worth. Each $S_{c}$ will be duplicated by weight $w_{c}$ addressing each corner’s distance to *P*. In an execution made by Gustavson [17];

(1) $$w_{c}={\left(max\left(\frac{1}{2}-\left(Dx_{c}^{2}-Dy_{c}^{2}\right),0\right)\right)}^{4}$$

where $Dx_{c}$ and $Dy_{c}$ are the distances in the *x* and *y* directions between each *c* and *P*. Only the *c* corners influence a point found inside a given Simplex, as other vertices in the Simplex matrix will disintegrate to zero preceding arriving at the current Simplex. Each edge of the Simplex is at an equivalent distance. The qualities are, as of this point, weighted depending on their connection to *P* to lessen the need to introduce. The outcome is the summation of weighted qualities for each N+1 corner of the current SN.

Rare examples are generally portrayed in the recurrence area, and a sign is controlled by indicating the plentifulness and stage for each recurrence [18]. Four-dimensional SN was used in this examination because of its low computational intricacies, low number of augmentations, low spot items, and scale-capacity for higher measurements [19,20]. SN execution utilized numerical organize changes and slope commotions to produce smooth and constant capacities without sharp edges.

Most VR games currently developed reuse the same assets when rendering the VR environment with NPCs and assets [21]. We used machine learning to generate 3D models employing a class of deep learning algorithms to address this shortfall and to ensure the cycling (user) did not become overly familiar with the environment being encountered. The objects were validated via the machine learning model and rendered via the Unity Game Engine.

The algorithm improved continuously by receiving feedback from the output mesh after every iteration. We used a UV map to use machine learning to optimize the game engine performance and avoid a lag when rendering objects. A standard map for the objects was created, and textures were applied on top of a base mesh and vertices of landmarks through the baking process. The algorithm generated new content based on provided examples as a draft for the designer to polish and finish the drafted content afterward.

PCG was fused with machine-learned models (PCGML) to generate VR content. The models were trained on existing VR contents [22]. PCGML is a technique for creating different content and replicating designed content to provide the player with infinite and unique gameplay variations. PCGML autonomously generates game artifacts at the time of generation. PCGML techniques were organized through two dimensions: Data Representation and Training Method. Data Representation is the underlying representation of the data used for training and generation. The machine learning technique was utilized for training the model. PCGML uses the created set of representative artifacts (NPCs, and objects in the scene) in the target domain as a model for the generator, and then the algorithm generates new content in this style.

### 2.2. Participants

Six participants aged 33.7 ± 8.5 years (mean ± SD) were recruited to the study through opportunistic sampling. The participants were approached from an opportunistic mixed-gender sample that participated in a previous study on visual search behavior in on-road cycling. Participants who volunteered to partake in the current study were male, self-classified as “experienced cyclist,” and cycled on average 3.8 ± 2.9 h per week. The Declaration of Helsinki’s tenants was observed, and institutional ethical approval for the study was provided. Before participation, written informed consent was obtained from the participants. MATLAB R2020a was used to analyze the data collected from the participants.

### 2.3. CHP-VR Simulator Procedure

The participants were invited (individually) to attend a laboratory that included the CHP-VR simulator set-up. On arrival, a demonstration of the equipment was provided. The stationary bicycle’s saddle and bar handle height was adjusted to suit the rider’s position, and the participant was made familiar with the bicycle gearing. It was clarified that the participant should cycle at a leisurely and comfortable speed, adhering to the cycling rules outlined in the Highway Code. Following the fitting and calibration of the head-mounted display (HMD), the participants completed an initial 3-minute practice trial. Following familiarization, a break was provided, and initial feedback about comfort was verified.

Participants were asked to cycle for five minutes in the VR environment (observing the Highway Code) within the second trial. VR visual search behavior (eye fixation and orientation) was recorded throughout the trial while the players anticipated (through speed adjustments and steering) other road users’ activities and the associated risks. The collected eye data incorporated the monocular pupillary distance (PD); the distance measured in millimeters between the centers of the pupils of the eyes [23]. Monocular PD refers to the distance between each eye and the bridge of the nose [24].

**Figure 1:**
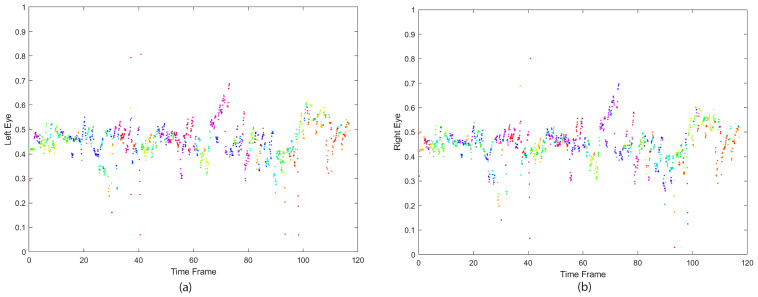
The orientation of the eyes while a player was interacting with the virtual world; collected from the HMD in the frontal plane. (**a**) Left eye. (**b**) Right eye.

HMD calibration started at the center of the screen, where the right and left eyes’ positions on the screen have an average distance equal to PD (mm). The adult’s average PD is estimated to be in the range of [54–74] (mm), while children’s PD range is [43–58] mm. The player’s PD was measured through the HMD’s initial calibration before starting the trial. Figure 1 displays a player’s left and right eye positions while incorporating the PD.

#### 2.3.1. FOVE VR Headset

**Figure 2:**
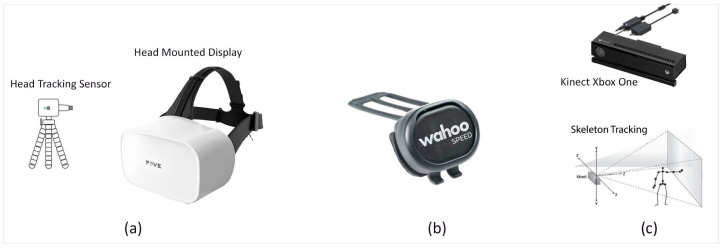
Sensors used in the development of the Cycling and Hazard Perception VR simulator. (**a**) FOVE VR Headset, (**b**) Wahoo RPM Cycling Speed Controller, and (**c**) Microsoft Kinect.

A FOVE HMD was used to develop the VR illustrated in Figure 2a. It was equipped with a position-tracking camera that recognizes the LED lights situated under the head-mounted cover with Inertial Measurement Units (IMU) and infrared (IR)-based framework eye-following sensors. The headset had a (90–100${}^{\circ }$) field of view (FOV), which gives a high-quality view experience. The HMD had a head position tracking sampling rate of 100 Hz. The showcase outline rate was 70 Hz, and the eye-following sensor had a 140–180 Hz sampling rate (120 fps). We positioned the position-following camera within three meters from the player while navigating the virtual environment. Adaptation of the VR headset enabled us to track and trace the player’s eyes position whilst interacting with the virtual assets and to determine the time to react to and avoid hazards.

#### 2.3.2. Wahoo RPM Cycling Speed

A low profile and wireless Wahoo RPM Speed Sensor was used to track the player’s cycling speed and transfer it to the virtual reality environment via a Bluetooth Smart system (https://uk.wahoofitness.com/devices/bike-sensors, accessed on 10 December 2017), (Figure 2b). The sensor uses a three-axis accelerometer to determine the wheel’s rotation, obtains a reference for the distance, and computes the wheel size. The sensor starts with self-calibration and enables the player to move forward in the scene based on the pedaling speed.

#### 2.3.3. Microsoft Kinect

The Kinect V2 device consists of an infrared laser-based IR emitter and a colored (RGB: red green blue) camera. It detected the position and orientation of 25 individual joints of the player and transferred it to the virtual reality environment displayed in Figure 2c. Body position was determined in a two-stage process; (I) computing a depth map and (II) inferring the participant’s body position. The depth map is constructed by analyzing a speckle pattern of infrared laser light [25]. Body parts are inferred using a randomized decision forest learned from over one million training examples [26].

**Figure 3:**
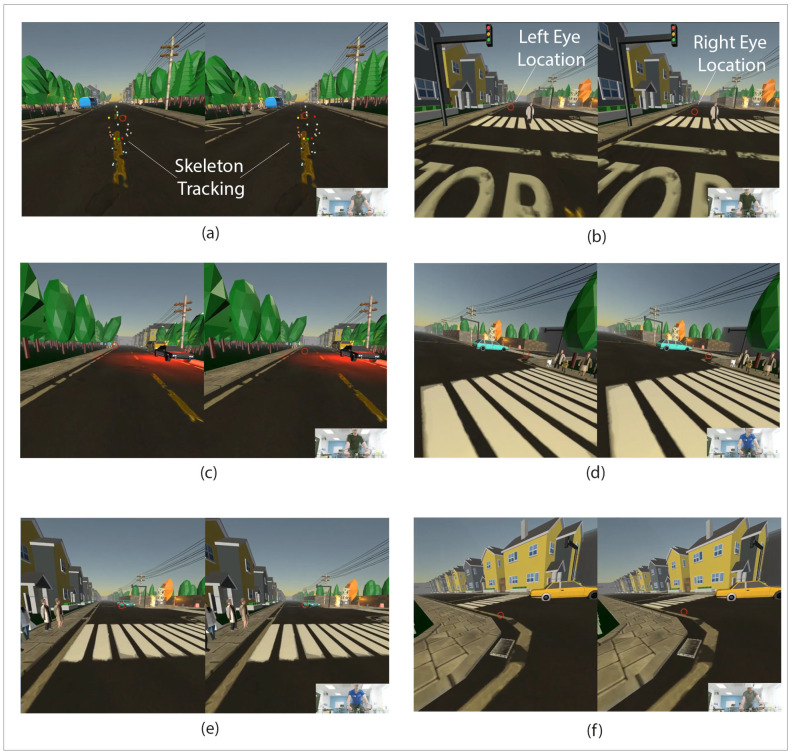
Cycling and Hazard Perception VR simulator scene with the left and right eye view through HMD. Eyes location in the scenes illustrated with red circles. (**a**) The player with the skeleton tracking cubes. (**b**) An NPC on the pedestrian cross. (**c**) A police car is passing on the opposite side of the cyclist’s path. (**d**) The cyclist turns to the right after crossing the pedestrians crossing and follows the car turning right. (**e**) Pedestrians are waiting to cross the pedestrian crossing. (**f**) The cyclist turns left while a taxi is leaving the junction.

Kinect provided us with approximately seventy skeleton joints (the body’s main joints and hand joints) and sixty frames per second (fps). The skeleton tracking is illustrated in Figure 3a by colored cubes showing the players’ main body joints. The static bike steering operation in real-time is transferred via the skeleton tracking system. This facilitates a braking system in the virtual world and maneuvering toward left, right, or straight ahead. Images in the right lower side of Figure 3 show players pictured while cycling on the static bike and wearing the VR headset collected via the Kinect.

### 2.4. Gaze Behavior in VR versus In Situ

To address presence by involvement [27], the current project compared the allocation of selective visual attention in the VR environment to an in situ environment. Evidence from perception-action coupling in the sports environment suggests that behavior, and consequently performance, in video-based experimental paradigms is distinct from in situ situations [28]. Furthermore, experimental task constraints influence the direction of visual attention; reduced perception–action coupling changes the direction of visual attention from the vision for action (control of movement) to the vision for perception (prediction of intention), [9]. After participating in the CHP-VR simulator, the participants’ visual search behavior was collected when cycling outdoors in situ; the experiment aimed to compare gaze behavior while cycling in an in situ environment to a VR environment.

#### In Situ Procedure

**Figure 4:**
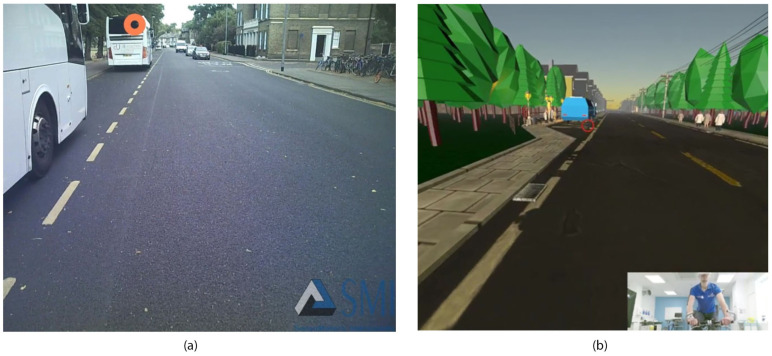
Gaze location based on Point of Regard (POR) on an Area of Interest (AOI) was collected (**a**) via an SMI eye tracker in situ and (**b**) via a head-mounted display in the VR environment.

The participants were asked to cycle both in the VR and in situ environments (on the road). For familiarity [29], for in situ testing, the participants were allowed to use their own bikes. In situ testing took place in daylight hours. Before commencing the ride, the route selection, procedures, and instructions (i.e., where to start and finish) were provided to participants. The selected route was a straight road in the center of Cambridge (UK). Next to the road’s left side was a park, and on the right side, a row of houses. The road was characterized by a selection of bus bays on the left of the route (i.e., in the direction of the traveling cyclist, see Figure 4a).

The participants were asked to cycle as they would typically, and throughout the entire trial, a research assistant followed the participant on a bicycle at a safe distance. The participant was required to wear a bicycle helmet to participate. A portable eye tracker was used to measure gaze behavior in on-road cycling. The SMI mobile eye gaze registration system (IviewETG, SensoMotoric Instruments Inc., Teltow, Germany, Ver. 1.0) consists of a pair of lightweight glasses with two infrared eye cameras and an HD scene camera. The SMI eye tracker measures the binocular direction of gaze via corneal reflection and the dark pupil principle. The center of the eye’s relative position concerning the scene camera is used to determine the direction of gaze in the environment with a frequency of 30 Hz and a spatial resolution of 0.1${}^{\circ }$ and gaze position accuracy of 0.5${}^{\circ }$.

The glasses were connected to a laptop (Lenovo X220, Thinkpad, Lenovo Group Limited, Beijing, China) stored in a backpack worn by the participant. The eye cameras have a gaze tracking range of 80${}^{\circ }$ horizontally and 60${}^{\circ }$ vertically where the high definition (HD) scene camera (1280 × 960 pixel, 24 Hz) has a tracking range of 60${}^{\circ }$ horizontally and 46${}^{\circ }$ vertically. The data were collected with Iview ETG software (Ver. 2.0, SMI, Teltow, Germany), and a three-point calibration in the sagittal plane at a five-meter distance from the participant was completed before the trial started. Following completion of the cycling route, the calibration of the eye tracker was checked.

**Figure 5:**
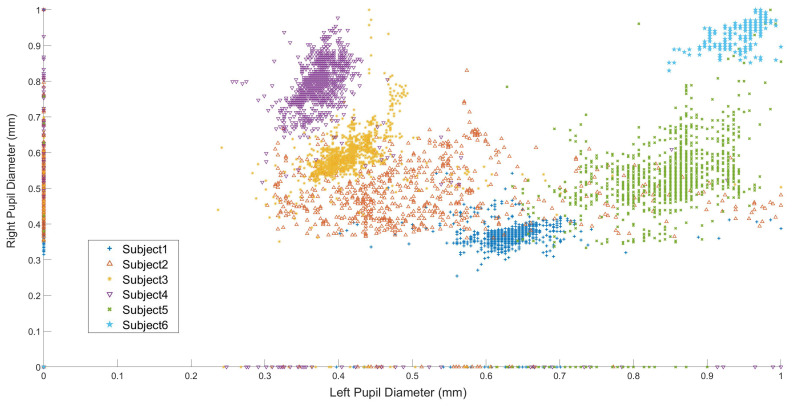
The normalized pupil diameter of subjects while passing the bus in situ taken from the SMI eye tracker.

**Figure 6:**
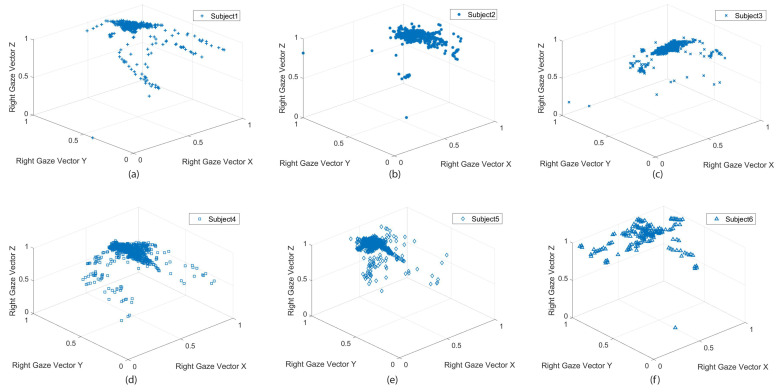
Subject’s normalized gaze vectors in three dimensions taken from the SMI eye tracker in situ. (**a**) Player number one, (**b**) player number two, (**c**) player number three, (**d**) player number four, (**e**) player number five and (**f**) player number six.

Figure 5 shows the participant’s normalized left and right eye pupil diameter size variation in situ while passing the bus. As illustrated in the Figure 6, subjects 2 (Figure 6b) and 5 (Figure 6e) had the most significant alteration in pupil diameter, while subjects 1 (Figure 6a), 3 (Figure 6c), 4 (Figure 6d), and 6 (Figure 6f) had a limited range of change. Figure 6 shows the subjects’ gaze vector and the visual search behavior in situ. The participants had different visual exploration performances although the trial’s conditions were the same. Some participants’ focus was more on the road ahead with an occasional visual search Figure 6b,c,e In contrast, others kept directing attention to objects and circumstances around them while cycling—a more comprehensive visual search span; Figure 6a,d,f.

## 3. Results

### Data Analysis

Scene selection was completed post data collection and consisted of identifying the most comparable scenarios identified in VR and in situ both within and between subjects. The most comparable identified scenes were characterized by passing a bus in a bus bay in VR and situ, as illustrated in Figure 4a,b. Gaze location was used to determine the gaze behavior based on the point-of-regard (POR) for an area of interest (AOI). A constant POR on an AOI indicated a fixation of gaze on this location. The gaze behavior in selected scenes (both VR and in situ) was analyzed from the first fixation of gaze on the parked bus until the cyclist passed the bus. The agreement between gaze behavior in VR and in situ was assessed via a Bland–Altman plot.

**Figure 7:**
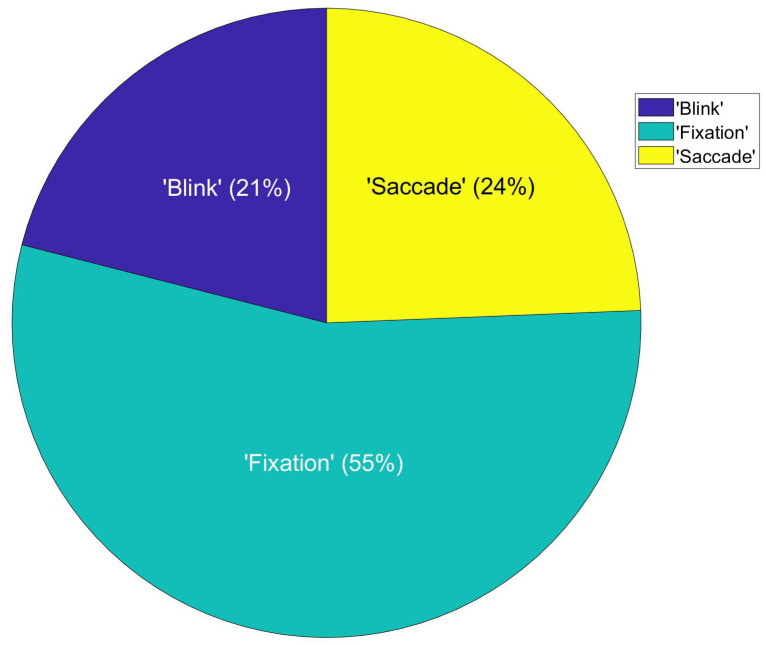
Example data from one participant, the percentage of saccades, blinks, and gaze fixation during the time of passing the parked bus in situ.

Figure 7 shows the percentage of saccades (24%), blinks (21%), and gaze fixation (55%) for a participant during the time of passing the parked bus in situ.

**Figure 8:**
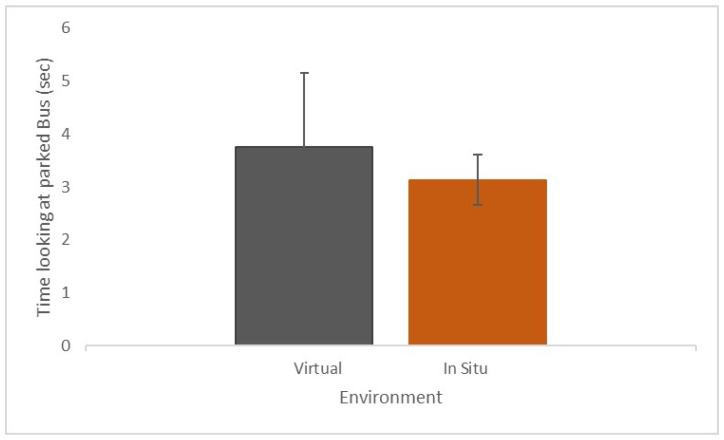
The time (sec) that gaze was directed toward the parked bus in VR (grey) and in situ (amber).

**Figure 9:**
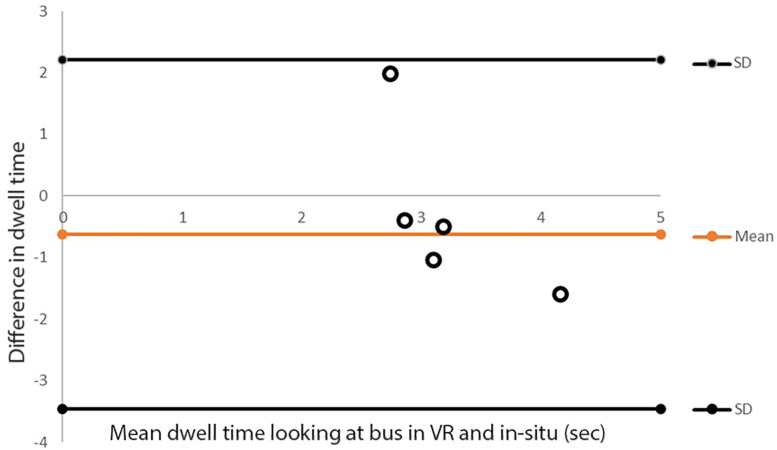
The limits of agreement between the dwell time at the bus in VR and in situ. The orange line represents the mean difference between VR and in situ. Horizontal black lines represent the 95% upper and lower limits (±1.96 SD).

Figure 8 present the average time that the gaze was directed to the bus in VR (3.7 s ± 1.4) and in situ (3.1 s ± 0.5). This difference had a medium effect size (d = 0.59), and 83% of the participants looked more at the parked bus in the VR environment than in situ. This suggests that participants tended to examine the bus longer in VR than in situ. To examine the agreement in dwell time at the bus, we conducted a limit of agreement analysis where it was identified that the average difference in dwell time between VR and in situ fell within the 95% confidence limits (see Figure 9).

Our initial observations for the VR and in situ eye-tracking recordings revealed that the number of assets in the VR environment was lower than in situ. For example, more cars were coming from the opposite direction, in addition to cars passing the cyclist in situ than in VR. These critical areas of interest are visually attended to (i.e., vision for movement), and thus cycling behavior can be adjusted accordingly. A recommendation is to enhance the number of used assets in VR based on observations in situ. This would drive the vision for movement and further enhance the immersive presence in VR. However, further studies with a larger sample size are needed to examine how presence is reflected in adopted gaze behavior.

## 4. Discussion

Cycling in traffic is a complex perceptual motor skill that requires cognitive abilities and strategic thinking. An ecological approach to examine the complex skill of cycling concerning hazard identification and perception requires the implementation of objective and subjective affordances (i.e., it should include opportunities for action based on the interactions between the cyclist and the environment). The current study describes the development of an egocentric 3D VR cycling simulator where cyclists can navigate and interact with a virtual environment. Pilot data collected on adults allowed perception–action coupling in VR and naturalistic environments to be compared by analyzing adopted gaze behavior.

The 3D VR cycling simulator development aimed to create a realistic alternative to overcome the challenges associated with examining hazard identification and perception whilst cycling in naturalistic environments. As such, we emphasized creating a presence in the VR environment over gamification. Presence in the VR environment was addressed (We regularly observed high levels of presence when participants made visual checks over the shoulder to look for traffic coming from behind) by including motor skill components (positioning on a bicycle, steering, creating speed through pedal motion, and braking with the brake levers), sounds related to motor vehicles, and ambient background traffic noise.

Concurrently, developed assets required strategic thinking in interacting with the environment (route choice, navigating cars, pedestrians, zebra crossings, and traffic lights). The 3D VR environment was developed around a PCG algorithm to increase the replay value, reduce development costs, increase adaptability, and save storage space. Using procedurally generated content accelerated the development workflow and included machine learning-based approaches to automatically generate content, infer constraints, and evaluate the output with limited human intervention [22]. Diversified NPCs and objects were created, combining the use of both procedural and machine learning-based strategies.

The idea’s primary motivation is to generate realistic scenarios that make the player feel more immersed in VR. This approach enabled us to assess the players’ behavior while interacting with the VR environment. Machine learning was used to train the model to learn the correlation between the input/output. This was done by creating a UV map where the normal map textures can be applied to the base mesh to produce new NPCs and objects. Continuous improvement of the learning algorithm enables detecting and repairing the generated objects.

To evaluate cycling in VR and in situ, we compared adopted gaze behavior concerning a specific scenario where the cyclist passed a parked bus. The length of time looking at the parked bus was not significantly different between the in situ and VR environments. However, 83% of participants looked for longer at the bus in VR compared to in situ (see Figure 8). Although the limits of agreement analysis indicated that differences fell within accepted confidence intervals, we have to acknowledge that the low number of participants influences the limits of agreement analysis.

Future work could explore gaze behavior in cycling and VR and in situ more to confirm the findings of [10] in a more dynamic environment. To examine this, it is recommended to design an in situ cycling course that can be replicated in VR. Observations within the analysis indicated some noticeable differences in the number of stimuli experienced. The in situ environment contained more and a greater variety of stimuli in comparison to the VR environment (see Figure 4), which would have served to attract visual attention away from the parked bus.

A recommendation is to adjust the procedurally generated unique and immersive content based on observations in naturalistic environments to create different levels of busyness on the road. Concurrently, there is a need to further validate in situ and VR cycling behavior with a larger sample size and to implement hazard perception features. An updated version of the simulator is under development where gamification and engagement are enhanced by implementing a scoring system.

**Figure 10:**
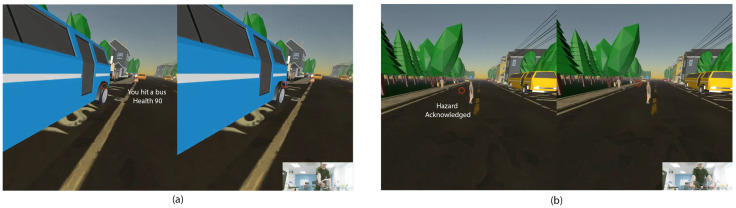
(**a**) The player hit a bus. (**b**) The player acknowledged the Hazard, and the NPC could cross the road safely.

Cyclists start with a score of 100 health and lose health upon failing to recognize hazards (e.g., not looking at dangers) or non-compliance with the road traffic rules. Figure 10a shows an example of a player colliding with a bus where the score is reduced. Figure 10b displays the player’s anticipated potential hazard, and the acknowledgment message that pops up on the screen. This update makes the simulator usable to train hazard identification and perception [8].

## 5. Conclusions

The developed CHP-VR simulator offers the opportunity to cycle in a virtual environment that reflects the situational demands and behavioral responses observed when cycling in naturalistic environments.

This allows participants to experience more complex situations in a safe and controlled environment whilst adjusting stimuli in a controlled manner (type, complexity, and amount of hazards). By including a gamification element, we can develop and implement scenarios where the situational awareness of hazards can be evaluated and trained. Further research is required to evaluate the effectiveness of hazard perception and situational awareness training.

## Data Availability

Not applicable.

## References

[1] Panter J.R., Jones A.P., Van Sluijs E.M., Griffin S.J. (2010). Neighborhood, route, and school environments and children’s active commuting. Am. J. Prev. Med..

[2] Transport Walking and Cycling Statistics, England. https://www.gov.uk/government/statistics/walking-and-cycling-statistics-england-2019.

[3] Panter J.R., Jones A.P., Van Sluijs E.M. (2008). Environmental determinants of active travel in youth: A review and framework for future research. Int. J. Behav. Nutr. Phys. Act..

[4] Groeger J.A., Chapman P. (1996). Judgement of traffic scenes: The role of danger and difficulty. Appl. Cogn. Psychol..

[5] Meyer S., Sagberg F., Torquato R. (2014). Traffic hazard perception among children. Transp. Res. Part Traffic Psychol. Behav..

[6] Tabei F., Askarian B., Chong J.W. (2020). Accident Detection System for Bicycle Riders. IEEE Sens. J..

[7] Igari D., Shimizu M., Fukuda R. (2008). Eye movements of elderly people while riding bicycles. Gerontechnology.

[8] Zeuwts L.H., Vansteenkiste P., Deconinck F.J., Cardon G., Lenoir M. (2017). Hazard perception in young cyclists and adult cyclists. Accid. Anal. Prev..

[9] Dicks M., Button C., Davids K. (2010). Examination of gaze behaviors under in situ and video simulation task constraints reveals differences in information pickup for perception and action. Atten. Percept. Psychophys..

[10] Drewes J., Feder S., Einhäuser W. (2021). Gaze During Locomotion in Virtual Reality and the Real World. Front. Neurosci..

[11] van Paridon K.N., Leivers H.K., Robertson P.J., Timmis M.A. (2019). Visual search behaviour in young cyclists: A naturalistic experiment. Transp. Res. Part Traffic Psychol. Behav..

[12] Hodgson C., Worth J. (2015). Research into the Impact of Bikeability Training on Children’s Ability to Perceive and Appropriately Respond to Hazards When Cycling on the Road. https://files.eric.ed.gov/fulltext/ED558729.pdf.

[13] Wirth M., Kohl S., Gradl S., Farlock R., Roth D., Eskofier B.M. (2021). Assessing Visual Exploratory Activity of Athletes in Virtual Reality Using Head Motion Characteristics. Sensors.

[14] Greuter S., Parker J., Stewart N., Leach G. Real-time procedural generation ofpseudo infinite’cities. Proceedings of the 1st International Conference on Computer Graphics and Interactive Techniques in Australasia and South East Asia.

[15] Greuter S., Parker J., Stewart N., Leach G. Undiscovered worlds–towards a framework for real-time procedural world generation. Proceedings of the Fifth International Digital Arts and Culture Conference.

[16] Perlin K. (1985). An image synthesizer. ACM Siggraph Comput. Graph..

[17] Perlin K. (2002). Improving noise. ACM Trans. Graph. (TOG).

[18] Lagae A., Lefebvre S., Cook R., DeRose T., Drettakis G., Ebert D.S., Lewis J.P., Perlin K., Zwicker M. (2010). A survey of procedural noise functions. Computer Graphics Forum.

[19] Lagae A. (2009). Wang Tiles in Computer Graphics. Synth. Lect. Comput. Graph. Animat..

[20] Cook R.L., DeRose T. (2005). Wavelet noise. ACM Trans. Graph. (TOG).

[21] Hendrikx M., Meijer S., Van Der Velden J., Iosup A. (2013). Procedural content generation for games: A survey. ACM Trans. Multimed. Comput. Commun. Appl. (TOMM).

[22] Summerville A., Snodgrass S., Guzdial M., Holmgård C., Hoover A.K., Isaksen A., Nealen A., Togelius J. (2018). Procedural content generation via machine learning (PCGML). IEEE Trans. Games.

[23] Millodot M. (2014). Dictionary of Optometry and Visual Science E-Book.

[24] McCleary D.S. (2018). The Optician Training Manual: Simple Steps to Becoming a Great Optician.

[25] Criminisi A., Shotton J., Konukoglu E. (2011). Decision forests for classification, regression, density estimation, manifold learning and semi-supervised learning. Microsoft Res. Camb. Tech. Rep..

[26] MacCormick J. (2011). How does the kinect work?. Present. Ved Dickinson Coll..

[27] Witmer B.G., Singer M.J. (1998). Measuring presence in virtual environments: A presence questionnaire. Presence.

[28] Dicks M., Davids K., Button C. (2009). Representative task design for the study of perception and action in sport. Int. J. Sport Psychol..

[29] Greene H.H., Rayner K. (2001). Eye movements and familiarity effects in visual search. Vis. Res..

